# Percutaneous Debulking of a Large Mobile Mitral Valve Vegetation Using the Angiovac

**DOI:** 10.7759/cureus.56537

**Published:** 2024-03-20

**Authors:** Nahush Bansal, Stephanie Younes, Mohammed Maaieh

**Affiliations:** 1 Department of Internal Medicine, The University of Toledo, Toledo, USA; 2 Department of Cardiology, ProMedica Toledo Hospital, Toledo, USA

**Keywords:** thrombi, mitral valve, infective endocarditis, cerebral protection system, angiovac debulking

## Abstract

The Angiovac aspiration system has been used successfully for the removal of intravascular material or thrombus in the right-sided heart structures, vena cava, implantable cardiac defibrillator, or other devices. For infective endocarditis, it is reserved for the patients who warrant but are not good candidates for the surgery. The evidence regarding Angiovac aspiration of the infective endocarditis of the left-sided heart valves is scarce. The risk of complications, including thrombi fragmentation leading to systemic embolization, damage to the cardiac structures and tissue, and hemodynamic instability, precludes the widespread use of this procedure, especially for the left-sided lesions. We report a case of successful removal of the mitral valve endocarditis using the Angiovac aspiration technique under the TEE guidance. A SENTINEL™ cerebral protection system was used to prevent embolus migration and a venous rather than an arterial access was used for reperfusion.

## Introduction

Infective endocarditis, with an incidence ranging from three to 10 cases per 100,000 population per year, contributes to extensive morbidity and mortality, especially if untreated [1]. Although medical therapy with guided antibiotic therapy can suffice in the management, some situations warrant urgent and necessary surgical intervention. These include development of acute heart failure due to valve dysfunction, presence of large vegetations (>10 mm) with a high risk of embolization, local tissue destruction, paravalvular infection and abscess, recurrent infections, and failed medical therapy [2]. Despite these scenarios, approximately 25% of patients are not referred to surgery mainly due to being at high surgical risk and poor prognosis, leaving them with little curative options [3].

The Angiovac system, consisting of an Angiovac cannula and extracorporeal Angiovac circuit, is a minimally invasive technique that has been successfully used to remove intravascular material like thrombi and emboli in the right heart and circulation [4-6]. The data regarding the use of this system in the left heart is scarce, and it is not yet FDA-approved for the same.

Percutaneous debulking of endocarditis is a rapidly evolving and upcoming field. Application to the left-sided chambers necessitates operator familiarity with the technology and comfort with left atrial procedures and embolic protection. Cerebral protection systems (TriGuard, Keystone Heart Ltd., Herzliya, Israel, SENTINEL™, Boston Scientific) are effectively used to reduce the embolization risk in transcatheter aortic valve replacement (TAVR) procedures, especially on the heavily calcified aortic valves [7].

We report a case in which a large mitral valve vegetation was successfully debulked in a very high surgical-risk patient using the novel Angiovac system and the SENTINEL™ cerebral protection system was deployed to prevent embolization and stroke.

## Case presentation

**Figure 1:**
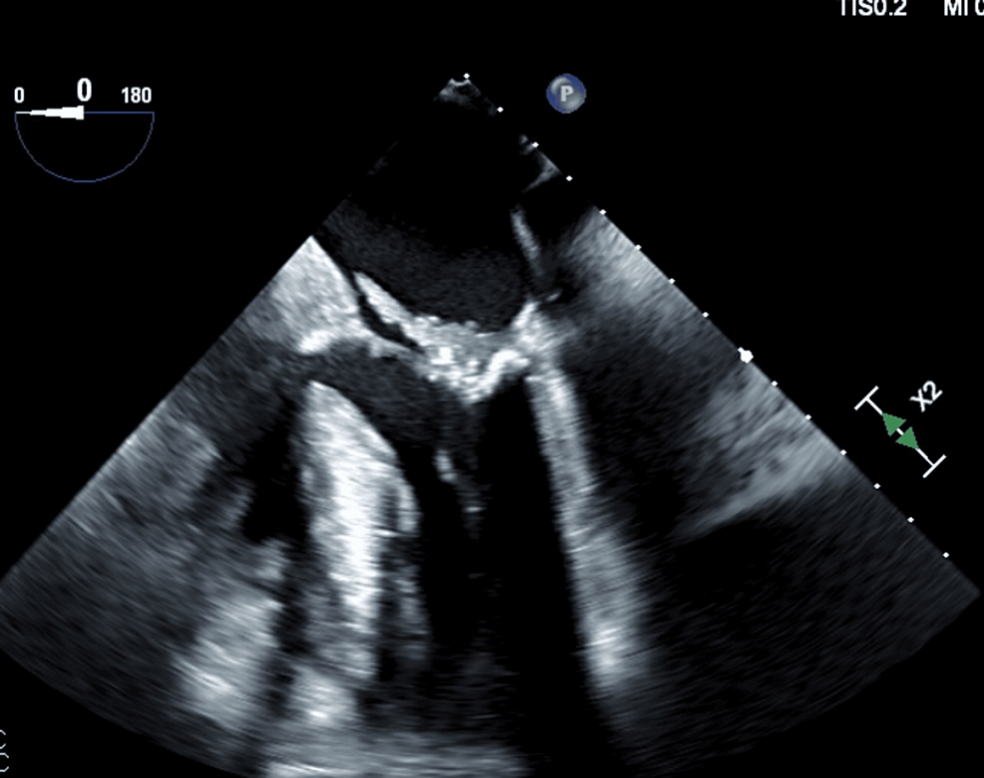
Transesophageal echocardiogram pre procedure showing a large vegetation on the posterior mitral valve leaflet

A 66-year-old female, a resident of a nursing home with a history of type 2 diabetes mellitus, hypertension, heart failure with preserved ejection fraction, chronic kidney disease on hemodialysis, Alzheimer’s dementia, with history of frequent falls, was admitted after witnessing a new mechanical fall and altered mental status. The patient was found to have an acute left hip comminuted intertrochanteric fracture on imaging. Further workup revealed positive blood cultures for methicillin-resistant *Staphylococcus lugdunensis*. A transthoracic echocardiogram showed concerns for endocarditis. Subsequent transesophageal echocardiogram (TEE) confirmed a very large mobile vegetation on the posterior mitral valve leaflet (Figure 1), measuring 3.3 cm in length, and moderate mitral regurgitation with early abscess formation. Given the comorbidities and mobility concerns with hip fracture, the patient was deemed at prohibitive risk for mitral valve replacement. Provided the significant risk of embolization from the large vegetation, a decision was made to debulk the vegetation using the Angiovac system.

**Figure 2:**
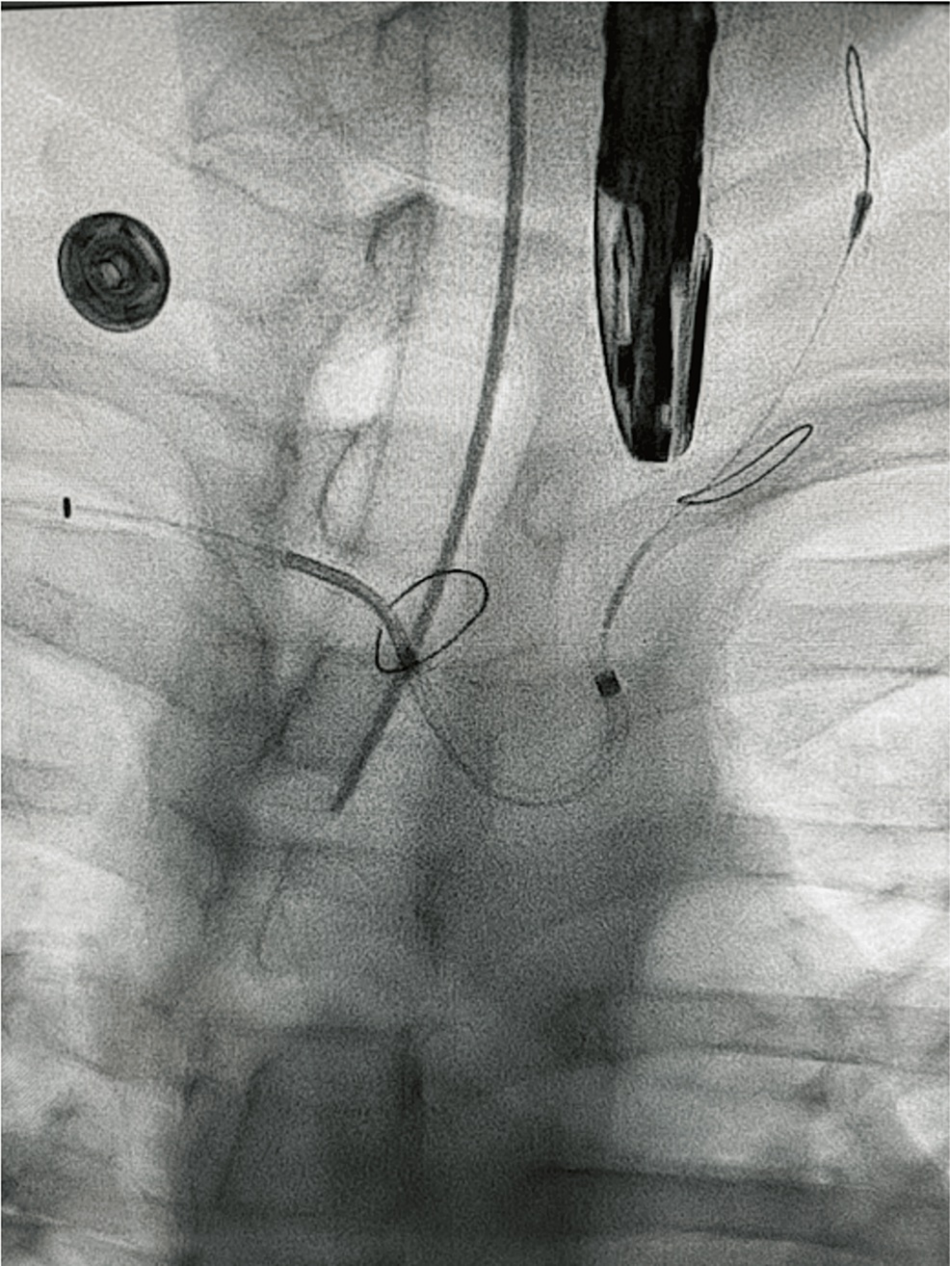
SENTINEL™ cerebral protection system deployed

Under TEE guidance, mitral valve vegetation debulking using the Angiovac system and SENTINEL™ cerebral protection system (CPS) device was performed. Access was obtained in the right and left femoral veins as well as the right radial artery. The aortogram revealed a bovine arch. Through the right radial access, CPS was deployed (Figure 2) in the left common carotid artery and the right innominate artery. Subsequently, the right femoral vein was serially dilated over a stiff wire and a 26F DrySeal sheath was placed. Similarly, the left femoral vein was upsized to a 17F return cannula for reinfusion. Full anticoagulation was administered using heparin.

**Figure 3:**
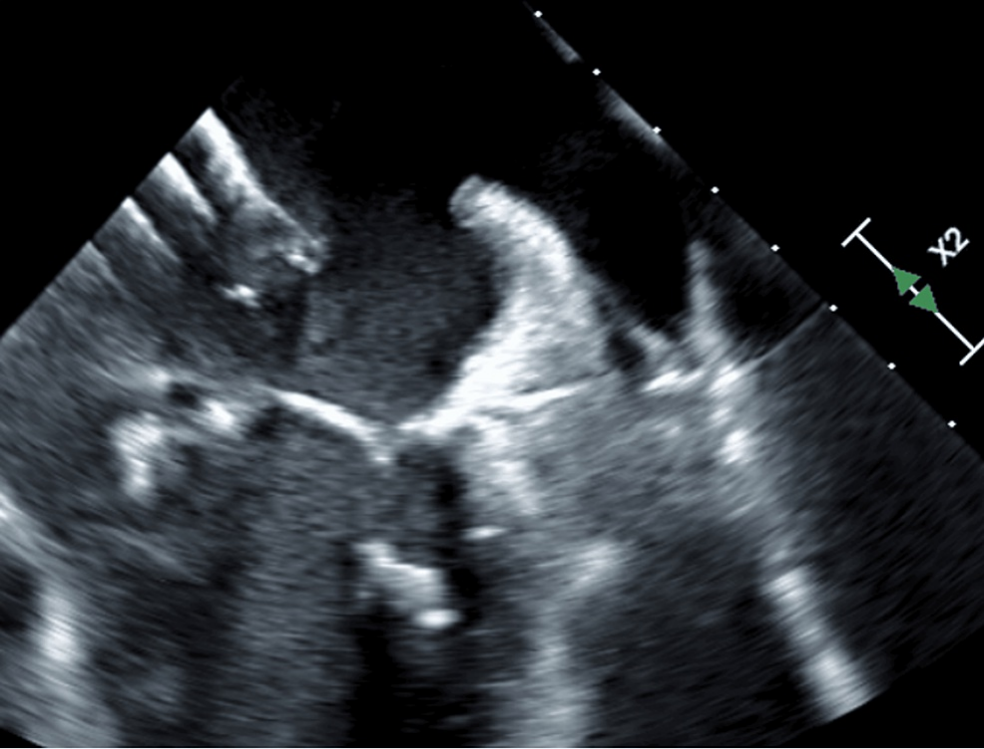
Transesophageal echocardiogram showing Angiovac cannula in the left atrium

**Figure 4:**
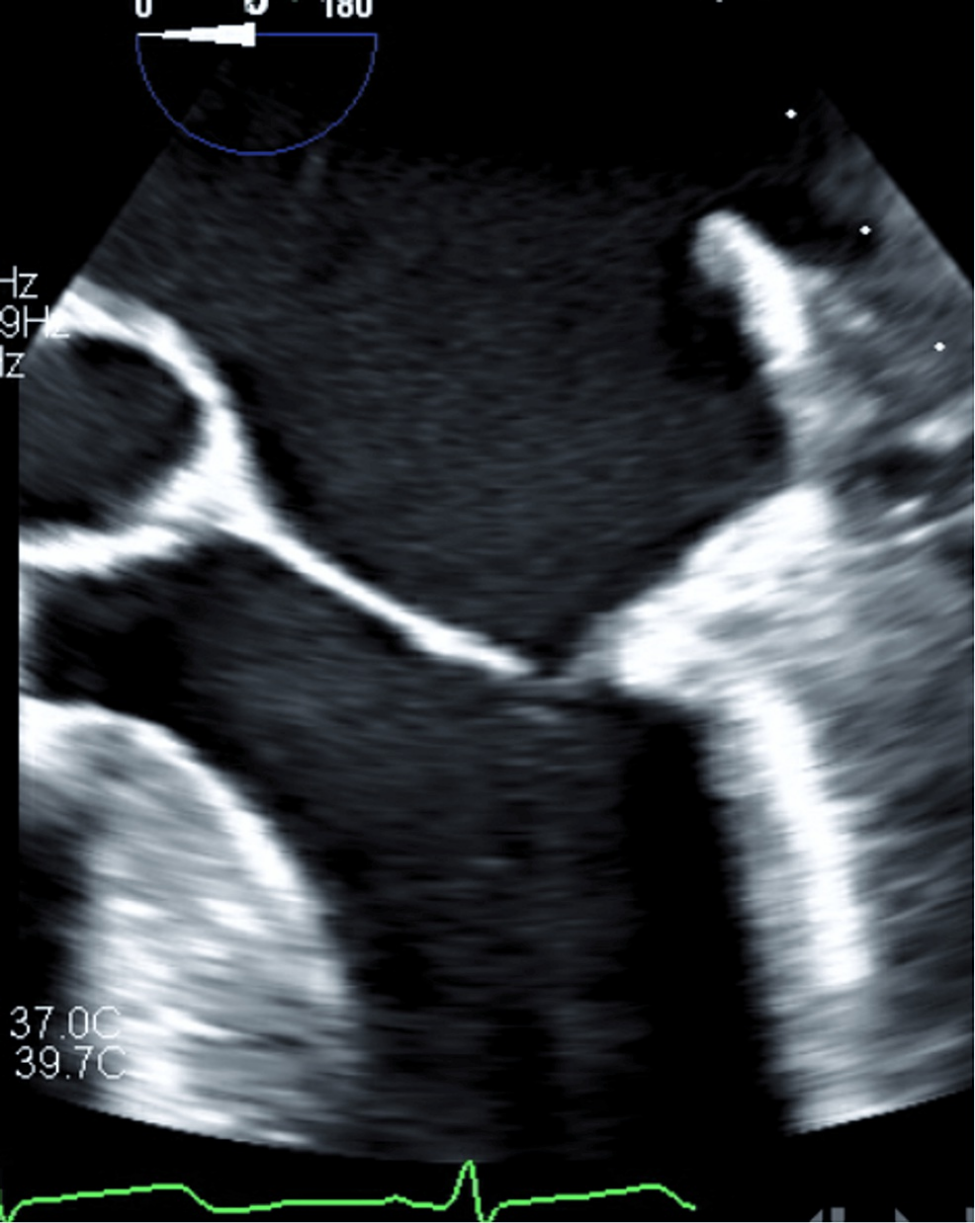
Transesophageal echocardiogram post procedure showing complete vegetation removal

**Figure 5:**
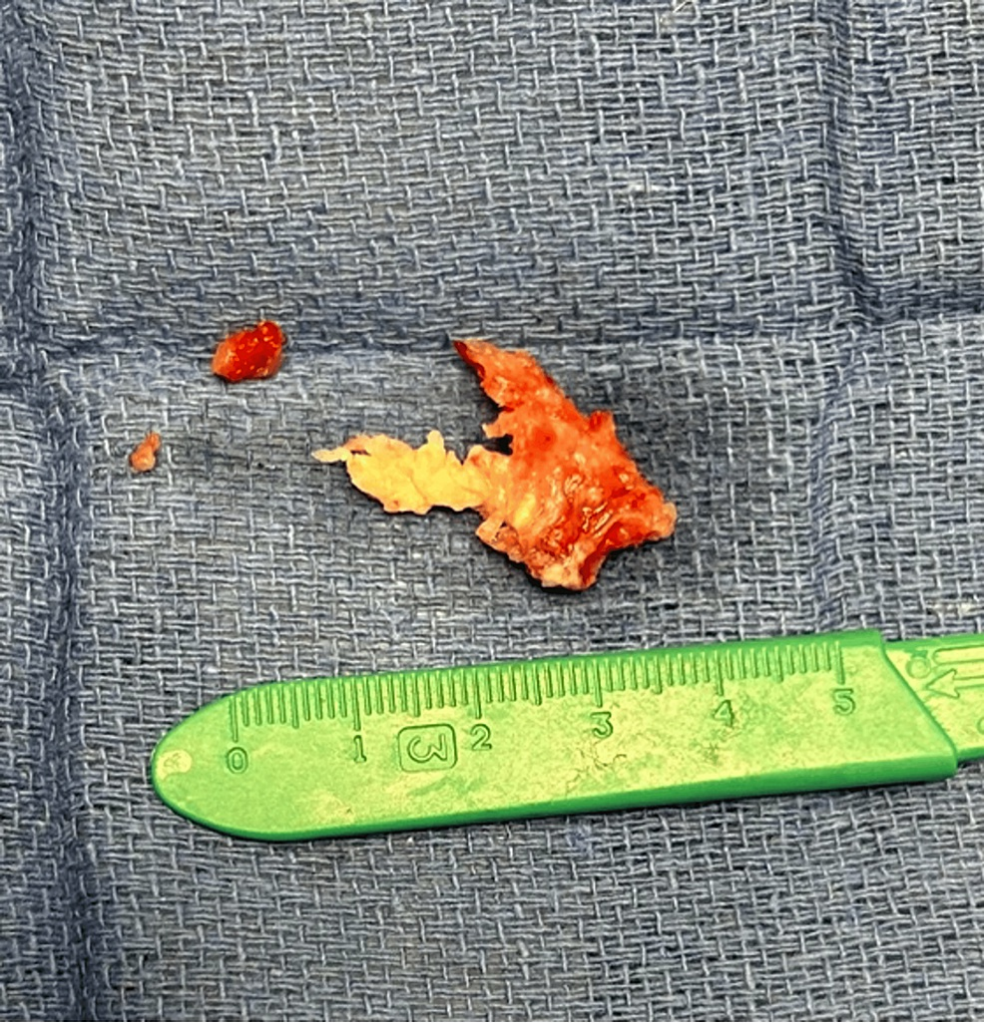
Aspirated thrombus

The transeptal SL1 sheath was advanced via the right femoral access and transseptal puncture was performed. The safe-sept wire was carefully advanced in the left upper pulmonary vein to avoid interactions with the mobile vegetation. This was then exchanged for a stiff wire and the septum was then dilated with a 14 mm balloon using balloon-assisted-tracking. The Angiovac cannula was taken into the left atrium (Figure 3). Flows through the extracorporeal bypass circuit were ramped up and the vegetation was aspirated under TEE guidance (Figure 4). The small residual vegetation was also removed subsequently, leading to the successful aspiration of the entire vegetation (Figure 5). The Angiovac cannula was pulled and the blood was returned via the left femoral vein and the tubing was then clamped. Figure eight stitches were placed at both femoral access sites. The CPS was retrieved with no debris in the filters. The patient remained hemodynamically stable throughout the procedure. She cleared her cultures and underwent hip surgery soon after.

## Discussion

The Angiovac system, consisting of a circuit and cannula to aspirate thrombi and emboli, has been used successfully to remove intravascular material in the right side of the heart, including tricuspid and pulmonic valve vegetations, vena cava, and implantable cardiac devices [4-8]. In clinical practice, this technique is primarily reserved as the treatment option for patients who are not surgical candidates. Registries have shown certain advantages of percutaneous debulking of tricuspid endocarditis as opposed to invasive surgical options, including less blood transfusion requirements, shorter hospital stays, and better recovery times [9]. One major benefit has been avoiding prosthetic implantation in patients who use intravenous drugs, as shown in a study by Veve et al. Their study demonstrated no significant differences in mortality and readmission between the groups undergoing percutaneous mechanical aspiration as opposed to valve surgery [10].

The use of an Angiovac system for the treatment of left-sided endocarditis is not FDA-approved. As this is an evolving field, the applications are expanding. The goal is to debulk the vegetation size to lower the embolic risk and microbial burden so that antibiotic therapy is more effective and potent. More data is required to establish the benefits of percutaneous debulking, however, in the meantime, this remains an option in experienced centers for patients who are not operative candidates.

The major risks of this procedure are embolization causing a stroke, damage to cardiac structures depending on the approach, and hemodynamic instability [11]. To reduce the risk of embolization to the brain, we used the SENTINEL^TM^ cerebral protection system in our patient. The cerebral protection system is an embolic protection system in which the filters are deployed to capture and remove thrombus or debris. It has been successfully used for TAVR procedures, especially in heavily calcified aortic valves [12]. Transesophageal echocardiogram guidance with the system allowed us to limit pump time. Given the system is flow-medicated with continuous aspiration, this limits interactions with the vegetation. We used venous access for reinfusion given her peripheral vasculature and this was well tolerated hemodynamically. Furthermore, as opposed to some previous cases in the literature for Angiovac removal of left-sided endocarditis, we used a transseptal rather than a transapical approach for the procedure [13].

## Conclusions

Angiovac aspiration, which has mainly been described in the removal of the thrombus or emboli in the right heart and circulation, can also be successfully used for debulking of large mobile mitral valve vegetations in patients with prohibitive risk of surgery. The risks of fragmentation causing systemic embolization can be mitigated by the use of the SENTINEL^TM^ cerebral protection system, device selection, as well as optimal imaging and visualization during the procedure. Venous access for reinfusion of blood is a safe option when the pump time is limited, with reduced risk of arterial access vascular complications
